# Global Analysis of Cell Wall Genes Revealed Putative Virulence Factors in the Dermatophyte *Trichophyton rubrum*

**DOI:** 10.3389/fmicb.2019.02168

**Published:** 2019-09-19

**Authors:** Maíra P. Martins, Larissa G. Silva, Antonio Rossi, Pablo R. Sanches, Larissa D. R. Souza, Nilce M. Martinez-Rossi

**Affiliations:** Department of Genetics, Ribeirão Preto Medical School, University of São Paulo, Ribeirão Preto, Brazil

**Keywords:** ambient stress, dermatophyte, drug targets, host-pathogen interactions, RNA-seq

## Abstract

The fungal cell wall is a structure in constant contact with the external environment. It confers shape to the cell and protects it from external threats. During host adaptation, the cell wall structure of fungal pathogens is continuously reshaped by the orchestrated action of numerous genes. These genes respond to environmental stresses and challenging growth conditions, influencing the infective potential of the fungus. Here, we aimed to identify cell wall biosynthesis-related genes that putatively encode virulence factors in *Trichophyton rubrum*. We used RNA-seq to examine the impact of two drugs, namely undecanoic acid, and acriflavine as well as the effects of the carbon source switching from glucose to keratin on *T. rubrum* cell wall metabolism. By using functional annotation based on Gene Ontology terms, we identified significantly differentially expressed cell wall-related genes in all stress conditions. We also exposed *T. rubrum* to osmotic and other cell wall stressors and evaluated the susceptibility and gene modulation in response to stress. The changes in the ambient environment caused continuous cell wall remodeling, forcing the fungus to undergo modulatory restructuring. The influence of the external challenges indicated a highly complex response pattern. The genes that were modulated simultaneously in the three stress conditions highlight potential targets for antifungal development.

## Introduction

The fungal cell wall directly interacts with the external environment and is vital to the survival of the organism. Its structural integrity is actively modulated in response to external and internal stresses. The cell wall supports fungal growth and development and enables the organism to endure hostile ambient conditions (Perlin, 2015; Valiante et al., 2015).

Pathogenic fungi use their cell walls to sense the host milieu and detect nutrients there. By modulating their cell wall components, fungal pathogens escape host immunity and invade the organism (Muszewska et al., 2017; Beauvais and Latge, 2018). Dermatophytes are fungal pathogens that infect keratinized tissues and dead epidermis in humans and animals. They produce several cell wall components that prevent them from being recognized by the host and function as virulence factors. Genome sequencing revealed that LysM binding domains are abundant in dermatophytes and probably mask pathogen cell wall components to confound the host immune response (Martinez et al., 2012; Kar et al., 2019). Also, the enrichment of several chitinase-encoding gene domains produces proteins supporting pathogen growth on a wide variety of substrates including soil and human skin. Thus, they enhance fungal infectiveness (Martinez et al., 2012). Differential protease secretion intensifies the inflammatory response induced by dermatophyte infection (Vermout et al., 2008). Moreover, through the production of extracellular vesicles the host innate immunity can be modulated (Bitencourt et al., 2018).

The constituents of the cell wall of the dermatophyte *Trichophyton rubrum* determine pathogen virulence. Mannans are associated with lymphocyte inhibition (Blake et al., 1991), a subtilisin homolog induces both immediate and delayed host immune responses (Woodfolk et al., 2000), and LysM proteins hide chitin and glucans from the host immune system (Kar et al., 2019). However, little is known about the relationships among dermatophyte pathogenesis, cell wall biosynthesis, and cell wall morphology.

Fungal virulence is determined by the coordinated expression of various genes mediating host-fungus interactions. Identification of the specific gene products or pathways crucial for fungal survival and infectiveness may potentially direct the development of new antifungal therapeutics (Bok et al., 2005; Martinez-Rossi et al., 2017). Changes in the ambient environment and external stressors continuously remodel the cell wall. Therefore, elucidation of the cell wall-related genes modulated in response to external challenges may disclose potential targets for antifungal drug development.

The cell wall is highly pertinent to antifungal drug discovery as it has a unique polysaccharide composition. For example, echinocandins inhibit β-(1,3)-*D*-glucan synthase biosynthesis in fungal cell walls. Thus, it is important to broaden our knowledge of fungal cell wall structure and the pathways regulating its formation (Grover, 2010; Perlin, 2015; Beauvais and Latge, 2018). Cell wall modulation in response to stressors may reveal putative targets for antifungal drug development (Martinez-Rossi et al., 2018).

The present study aimed to establish the modulation profile of cell wall genes in response to ambient challenges in the dermatophyte *T. rubrum*. We evaluated RNA-seq results under stress conditions wherein the pathogen was exposed to two drugs, or forced to switch its carbon source from glucose to keratin. Keratin mimics the host environment, and the drugs chosen, acriflavine and undecanoic acid, present a non-specific antifungal activity against *T. rubrum.* We identified a wide range of genes putatively encoding the virulence factors of *T. rubrum*. Those genes whose expression levels were altered under all stress conditions were considered as possible targets for antifungal drug development. We also challenged *T. rubrum* with various osmotic and cell wall stressor agents and evaluated their effects on the modulation of different genes controlling cell wall morphology.

## Materials and Methods

### *T. rubrum* Strain and Culture Conditions for RNA-seq

*T. rubrum* strain CBS118892 (*Centraalbureau voor Schimmelcultures*, Netherlands) was maintained on malt extract agar (MEA; 2% (w/v) glucose, 2% (w/v) malt extract, 0.1% (w/v) peptone, pH 5.7) for 17 d at 28°C. Approximately 10^6^ conidia mL^–1^, obtained as previously described (Fachin et al., 1996; Jacob et al., 2012), were inoculated into 100 mL Sabouraud dextrose broth (SDB; 2% (w/v) glucose, 1% (w/v) peptone) and incubated at 28°C for 96 h under agitation; one flask was used for each time point of each tested condition (keratin or glucose). The mycelia in each flask were aseptically filtered and transferred into a new flask containing 100 mL minimal medium (MM) at pH 5.0 (Cove, 1966) supplemented with 70 mM nitrate (Sigma Aldrich Corp., St. Louis, MO, United States) and either 50 mM glucose (Sigma Aldrich Corp., St. Louis, MO, United States) (control) or 0.5% (w/v) bovine keratin (treatment) as the carbon source. All experiments were performed in three biological replicates. The cultures were incubated under agitation at 28°C for 24 h, 48 h, or 96 h. Mycelia were collected and the RNA was extracted from them. Drug-related library data are available in the GEO database under accession nos. GSE102872 and GSE40425; these results were obtained through the exposure of 96 h-grown SDB mycelia to 1.75 μg mL^–1^ acriflavine (ACF; Sigma Aldrich Corp., St. Louis, MO, United States) (Persinoti et al., 2014) or 17.5 μg mL^–1^ undecanoic acid (UDA; Sigma Aldrich Corp., St. Louis, MO, United States) (Mendes et al., 2018) in RPMI 1640 medium (Thermo Fisher Scientific, Waltham, MA, United States). *T. rubrum* susceptibility, as previously described, was determined by assessing MIC using the microdilution approach (M38-A) proposed by the Clinical and Laboratory Standards Institute (CLSI) (Persinoti et al., 2014; Mendes et al., 2018).

### Stress Conditions

Susceptibility of *T. rubrum* to stressors was evaluated using plates containing SDB medium (1.5% (w/v) agar) supplemented with KCl (0.5 M), NaCl (0.5 M), SDS (0.01% (w/v)), sorbitol (1.2 M), Congo Red (CR; 200 μg mL^–1^), or Calcofluor White (CFW; 200 μg mL^–1^). Radial growth was measured using the diameter of propagated mycelia in centimeters on the 6th day of development. Data correspond to the means of three measurements. For qRT-PCR, mycelia grown for 96 h in flasks containing SDB were filtered, transferred, and incubated for 1 or 3 h in SDB containing the afore mentioned stressor agents. After incubation, mycelia from each experiment were frozen in liquid nitrogen, stored at −80°C, and used for RNA extraction.

### RNA Isolation, Sequencing, and Data Analysis

Total RNA was isolated from ∼100 mg mycelia using an Illustra RNAspin mini isolation kit (GE Healthcare, Chicago, IL, United States). The RNA concentrations were determined using a NanoDrop ND-1000 spectrophotometer (Thermo Fisher Scientific, Waltham, MA, United States). RNA quality was validated with an Agilent 2100 bioanalyzer (Agilent Technologies, Santa Clara, CA, United States). Equal amounts of RNA from three independent biological replicates of *T. rubrum* keratin/glucose cultures at 24, 48, or 96 h were used for the synthesis of cDNA with the TruSeq RNA library Kit (Illumina, San Diego, CA, United States), and sequenced with a Hiseq 2000 sequencer (Illumina, San Diego, CA, United States) according to the manufacturer’s instructions. Paired-end reads 150 bp in size were generated. Raw read data obtained via RNA-seq were filtered for quality control by FastQC tool and trimmed with Trimmomatic (Bolger et al., 2014) to remove adapters and Illumina-specific sequences. Trimmed paired-end reads from each sample were aligned to the *T. rubrum* reference genome^1^ with STAR aligner (Dobin et al., 2013). Gene-level read counts were quantified with STAR’s ‘-quantModeGeneCounts’ parameter. Differential expression was analyzed with the DESeq2 Bioconductor package (Love et al., 2014). A Benjamini-Hochberg correction (Benjamini and Hochberg, 1995) adjusted the *P* threshold and was applied to reveal statistically significant changes in gene expression levels. It was set to 0.05, with a log_2_ fold change ± 1.5 and postulated as a significantly modulated transcript abundance level. Genes surpassing these thresholds are hereinafter referred to as differentially expressed genes (DEG). They were functionally categorized with the Gene Ontology (GO) terms assigned by the Blast2GO algorithm (Robinson et al., 2011; Thorvaldsdottir et al., 2012). Highly represented categories were determined by enrichment analysis with the BayGO algorithm (Vencio et al., 2006). After functional annotation analysis, the cell wall-related genes in *T. rubrum* RNA-seq were identified with the R script mapping modulated Genes and Gene Ontology terms. A customized R script detected the descendant terms of the GO cellular component ‘cell wall’ with the Bioconductor GO.db package (Carlson, 2018). The genes are listed in Supplementary Table S1. Nine of these genes were arbitrarily selected to validate the results obtained through RNA-seq.

### cDNA Synthesis, and qRT-PCR Analysis

Total RNA was treated with DNase I (Sigma Aldrich Corp., St. Louis, MO, United States) to remove residual genomic DNA. The complementary DNA (cDNA) was generated with a high-capacity cDNA reverse transcription kit (Applied Biosystems, Foster City, CA, United States). The qRT-PCR was performed with a StepOnePlus Real-Time PCR system (Applied Biosystems, Foster City, CA, United States). Gene expression was analyzed to confirm keratin RNA-seq and evaluate transcriptional responses to osmotic- and cell wall stressors. Reactions were run in a total volume of 12.5 μL with Power SYBR Green PCR Master Mix (Applied Biosystems, Foster City, CA, United States) and 50 ng template cDNA. Primer sequences, concentrations, and reaction efficiencies are listed in Supplementary Table S2. Glyceraldehyde-3-phosphate dehydrogenase (*gapdh*) and DNA-dependent RNA polymerase II (*rpb 2*) were used as internal controls (Jacob et al., 2012). Data were derived from three independent replicates. The 2^–ΔΔct^ relative expression quantification method was used to calculate gene responsiveness (Livak and Schmittgen, 2001). Data were statistically analyzed with Student’s *t*-test (RNA-seq validation) or one-way ANOVA followed by Tukey’s *post hoc* test. The statistical software was GraphPad Prism v. 5.1 (GraphPad Software, La Jolla, CA, United States).

## Results

### Global DEG Identification in Response to Keratin

For comprehensive analysis of the *T. rubrum* genes expressed in response to keratin, we performed high-throughput sequencing (RNA-seq) using glucose as the control. Approximately 192 million high-quality reads generated ∼167 million mapped paired-end sequences (Supplementary Table S3). The dataset comprised 2,797 genes that were modulated in response to keratin relative to glucose. The upregulated and downregulated transcripts were defined using a 1.5-fold change cutoff (≥ 2.8-fold difference and a stringent statistical significance threshold of *P* < 0.05). We used the Blast2Go tool (Gotz et al., 2008) to depict the functional distribution of the modulated genes. This information elucidated the functionality of the DEGs in response to changing growth conditions.

### Identification of the Genes Associated With Cell Wall Synthesis in the RNA-seq Libraries

**FIGURE 1:**
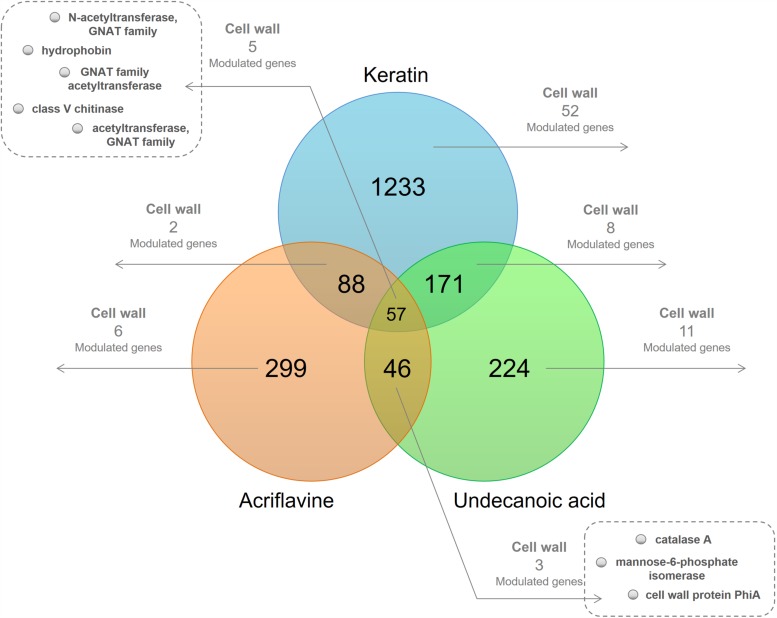
Venn diagram of the differentially expressed genes (DEGs) in the libraries. At the intersections, number of genes modulated in at least one of the time points of each of the three libraries. The number of modulated cell wall-related genes present in at least one of the time points are highlighted.

We used functional annotation based on GO terms to identify cell wall-related genes among the DEGs for the keratin, acriflavine, and undecanoic acid RNA-seq (Figure 1 and Supplementary Table S1). The R script found cell wall-related genes by detecting direct child terms of the cellular component ‘cell wall’ in GO.

We identified genes common to all three libraries and those that were common to two of them. We also determined those unique to each library. We obtained 20 downregulated and 47 upregulated genes in response to keratin culture. Acriflavine treatment induced five genes and repressed 12 others. There were 19 downregulated and nine upregulated genes in response after undecanoic acid exposure. Five of the genes modulated in response to acriflavine were common to all three libraries. Four were altered after acriflavine undecanoic acid treatment. Two others changed after exposure to acriflavine keratin (Supplementary Table S1).

### Genes Modulated in Response to Acriflavine

Acriflavine treatment repressed acetyltransferases of the GNAT (Gcn5-related *N*-acetyltransferase) family (TERG_02517, TERG_05545, and TERG_07408) and induced genes essential for cell wall resistance such as catalases (TERG_01252, TERG_06053) and the hydrophobin (TERG_04234) (Supplementary Table S1 and results extracted from Persinoti et al., 2014).

### Genes Modulated in Response to Undecanoic Acid

Undecanoic acid treatment upregulated genes encoding glycosyl hydrolases (TERG_12281, TERG_12282, TERG_06016), a cytosolic Cu/Zn superoxide dismutase (TERG_08969), and catalase A (TERG_01252). The latter two were also induced by acriflavine exposure.

Undecanoic acid also downregulated the cell wall glucanase *scw11* (TERG_05576), genes encoding chitinases (TERG_05626, TERG_05625, and TERG_02350), and other cell wall-associated genes (TERG_03624, TERG_06144, and TERG_07456). Both undecanoic acid and acriflavine downregulated genes encoding three GNAT family *N*-acetyltransferases (TERG_02517, TERG_05545, and TERG_07408). Whereas undecanoic acid downregulated the gene encoding hydrophobin (TERG_04234), acriflavine upregulated it (Supplementary Table S1 and results extracted from Mendes et al., 2018).

### Genes Modulated in Response to Keratin

The shift from culture media containing glucose to those with keratin induced significantly more genes than it repressed. Media with keratin downregulated certain genes encoding GNAT family acetyltransferases (TERG_05545, TERG_07987, TERG_08211, and TERG_07408), and the Wiskott-Aldrich syndrome protein family member 2 (TERG_00693).

Genes encoding glycosyl hydrolases (TERG_05530, TERG_01837, TERG_02742, and others), glucanases (TERG_04887 and TERG_07817), chitinases (TERG_05626 and TERG_06925), and an alpha-1,2-mannosyltransferase (TERG_06397) were upregulated in response to keratin. Hydrophobin (TERG_04234) overexpression was observed as it was for the acriflavine treatment.

### Osmotic and Cell Wall Stressors Influence *T. rubrum* Development

**FIGURE 2:**
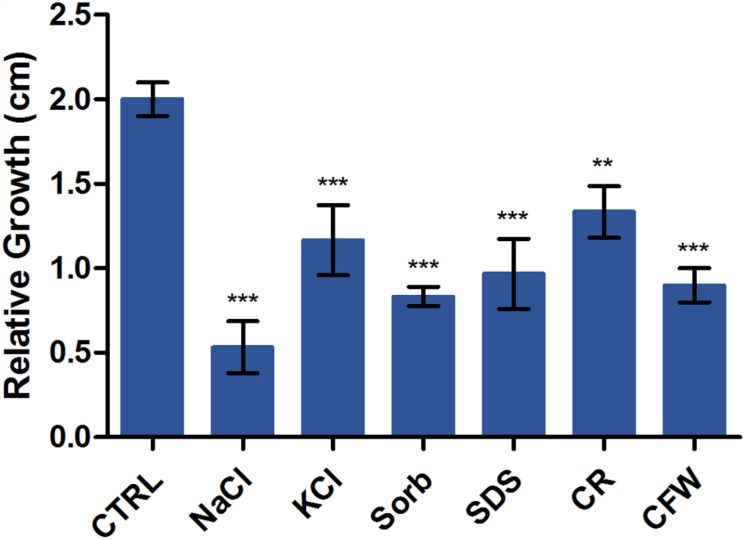
Susceptibility of *T. rubrum* to various stressors. Strains were inoculated on Sabouraud dextrose broth with or without the stressors NaCl, KCl, sorbitol (Sorb), SDS, Congo Red (CR), and Calcofluor White (CFW) at 28°C for 6 d. The graph represents the relative colony diameters (in centimeters) of each strain in the presence of the stressors relative to the control (CTRL), without the stressors. Data are means of three biological replicates. The error bar indicates the standard deviation (SD). Asterisks indicate statistical significance determined by ANOVA followed by Tukey’s *ad hoc* test (^∗∗^*P* < 0.01; ^∗∗∗^*P* < 0.001).

We evaluated the relative sensitivities of *T. rubrum* grown on SDB medium with or without KCl, NaCl, SDS, sorbitol, CR, and CFW. Figure 2 shows radial colony growth inhibition induced by each stressor. A qRT-PCR analysis evaluated the effects of these stressors on the transcription profiles of STE/STE20/YSK protein kinase *kic1* (TERG_01721), cell morphogenesis protein *sog2* (TERG_07599), conidiophore development protein *hym1* (TERG_00759), AGC/NDR/NDR protein kinase *cbk1* (TERG_03379), kinase activator protein *mob2* (TERG_02863), cell morphogenesis protein *tao3* (TERG_01788), 1,3-β-glucan synthase component *fks* (TERG_01127), chitin synthase 2 *chs* (TERG_12319), class III chitinase *cts* (TERG_02705), and cell wall glucanase *scw11* (TERG_05576).

### RNA-Seq Validation by qRT-PCR

**FIGURE 3:**
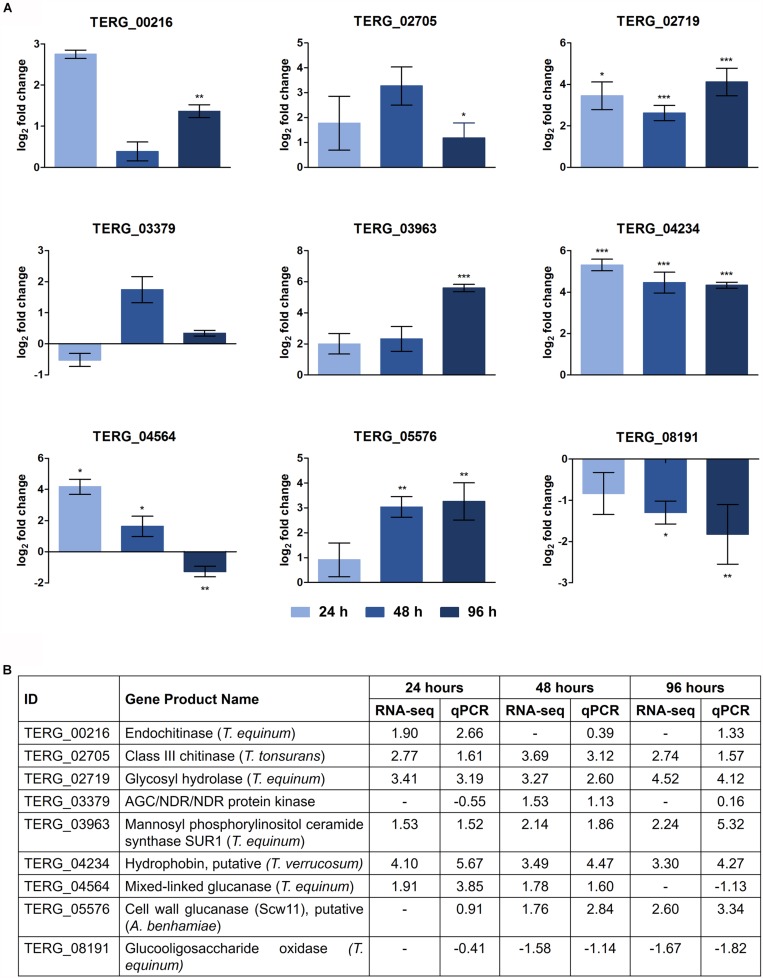
Validation of DEGs under keratin cultivation by qRT-PCR. **(A)** Gene expression levels are represented as log_2_-fold changes relative to each control condition (24 h keratin × 24 h glucose; 48 h keratin × 48 h glucose; 96 h keratin × 96 h glucose). Asterisks indicate statistical significance determined by Student’s *t*-tests comparing treatment and control conditions at each time point (^∗^*P* < 0.05; ^∗∗^*P* < 0.01; ^∗∗∗^*P* < 0.001). **(B)** Comparison of gene expression levels determined by RNA-seq with those evaluated by qRT-PCR (Pearson’s correlation, *r* > 0.82; *P* < 0.001).

The DEGs selected and assayed through qRT-PCR validated the RNA-seq results. The expression patterns were observed in the samples, examined in triplicate, and confirmed the reliability of the RNA-seq results (Pearson’s correlation, *r* > 0.82; *P* < 0.001) (Figure 3).

## Discussion

### The Cell Wall Is a Virulence Factor Modulated in Response to Environmental Conditions

The fungal cell wall is the most promising virulence factor target for drug discovery because of its location, mediation of fungal-host interactions, uniqueness of composition, and absolute necessity for the survival of the pathogen. The roles of the cell wall in adhesion, colonization, signaling, and immune recognition make it vital for pathogen infection (Arana et al., 2009; Hasim and Coleman, 2019). As cell wall of fungi enables them to interact dynamically with the ambient environment, evaluation of the genes responsive to environmental stresses may lead to a better comprehension of the role of the cell wall in *T. rubrum*.

### Only a Few Cell Wall-Related Genes Were Modulated in Response to Acriflavine

*T. rubrum* was subjected to sublethal doses of acriflavine which has antiseptic and anticancer properties but whose use is limited by its toxicity (Persinoti et al., 2014). RNA sequencing analysis revealed modulation of only a few cell wall-related genes. Thus, acriflavine had minimal influence on *T. rubrum* cell growth, shape, or protection.

Two catalase genes were upregulated in response to acriflavine. Acriflavine resistance was observed in *Dictyostelium*, as a result of catalase A gene (*catA*) disruption. This gene has peroxidase activity that modifies acriflavine into its cytotoxic form (Garcia et al., 2002). Catalases protect cells against peroxide-induced damage. They are also essential for pathogen virulence and cell wall integrity during host invasion (Skamnioti et al., 2007). The observed upregulation of two catalases in *T. rubrum* suggests that the pathogen metabolized the drug and induced catalase during cell wall formation and/or maintenance in the attempt to increases its virulence.

Hydrophobin is a small cysteine-rich protein secreted only by filamentous fungi. It regulates cell wall integrity (Wosten, 2001; Mankel et al., 2002) and is upregulated in response to acriflavine exposure. Acriflavine increases hydrophobicity, decreases hydrophilicity, induces impermeability, and alters cell wall thickness (Glynn and Priest, 1970; Kawai et al., 2009). Hydrophobins are amphiphilic and lower the surface tension of water (Linder et al., 2005). The hydrophobin induction detected in *T. rubrum* in response to acriflavine exposure suggests that the fungus was attempting to counterbalance the increase in cell wall hydrophobicity caused by the drug.

Only a few genes are repressed in response to acriflavine including three members of the GNAT family of acetyltransferases. They are involved in post-translational modification by transferring acetyl groups from acetyl-CoA to their cognate substrates (Favrot et al., 2016). GNATs regulate transcriptional responses to various environmental stressors such as heat, cold, oxidative stress, and low nutrient availability (Huisinga and Pugh, 2004; O’Meara et al., 2010). The GNAT repression observed in our results suggests increasing stress-mediated activation and drug susceptibility in *T. rubrum*.

### Undecanoic Acid Mainly Represses *T. rubrum* Cell Wall-Associated Genes

Undecanoic acid exposure, especially for 3 h, downregulated several genes associated with cell wall morphogenesis, including *scw11*, which encodes the cell wall-based endo-1,3-β-glucanase (Millet et al., 2018), the SUN domain protein Uth1-like, encoding a β-(1,3)-glucan-hydrolyzing enzyme (Gastebois et al., 2013), a cell wall serine-threonine-rich galactomannoprotein Mp1 (Gautam et al., 2011), and a cell wall structural protein PhiA. Repression of genes related to cell wall maintenance was reported for *Aspergillus fumigatus* exposed to the antimalarial drug artemisinin, which is known to have antifungal activity (Gautam et al., 2011). This same effect was also reported in *T. rubrum* subjected to ketoconazole and amphotericin B (Yu et al., 2007). It was shown that the antifungal agent undecanoic acid effectively impairs *T. rubrum* cell wall formation (Mendes et al., 2018).

Undecanoic acid represses GNAT family *N*-acetyltransferase genes, similarly to what was observed for acriflavine. The gene encoding a hydrophobin, upregulated by acriflavine was downregulated in response to undecanoic acid exposure. Undecanoic acid damages the cell wall of *T. rubrum* by reducing its ergosterol content and altering its fatty acid metabolism (Mendes et al., 2018). Ergosterol controls water penetration and regulates enzymes involved in protein transport and chitin synthesis. Excessive intracellular water uptake may occur when ergosterol metabolism is deregulated. The hydrophobin gene appears to be involved in the water flux across the fungal cell wall (Abe et al., 2009; Zuza-Alves et al., 2017) and is associated with fungal pathogenesis, mediating the attachment of fungal infective structures to their targets (Wosten, 2001). Undecanoic acid exposure in *T. rubrum* may have negatively impacted its pathogenicity by downregulating hydrophobins.

The gene encoding a class V chitinase (glycoside hydrolase family 18 protein-GH18) and another encoding an endochitinase were repressed by undecanoic acid. As chitin is absent in mammalian cells and essential for fungal cell wall integrity, it is a promising target for antifungal drug development (Ruiz-Herrera and San-Blas, 2003). The observed repressive effect against chitinases emphasize the promising use of undecanoic acid in fungal treatment. Acriflavine also repressed the GH18; therefore, both drugs may disrupt important biochemical events involved in the establishment and maintenance of fungal infection in the host (Persinoti et al., 2014). In *T. rubrum*, this chitinase presents LysM domains and may be involved in keratin degradation (Lopes et al., 2019).

Three glycosyl hydrolase genes were upregulated in response to undecanoic acid. The TERG_06016, identified as a PHO system negative regulator, responsible for the hydrolysis of O-glycosyl compounds, and two cell wall acid trehalases, that shelter fungi against several physiological and environmental stressors (Arguelles, 2000). These inductions were detected 3 h after undecanoic acid treatment. Thus, *T. rubrum* immediately responded to this exposure and attempted to offset the damage caused by the stress in the cell walls. We also observed that the genes improving fungal stress tolerance were upregulated after 12 h incubation. These included the cytosolic Cu/Zn superoxide dismutase and catalase A. Moreover, cell wall formation was repressed.

### The Cell Wall Is Actively Synthesized When *T. rubrum* Is Cultured With Keratin

We supplied *T. rubrum* with keratin to mimic the initial host infection stages. Mycelia harvested after 96 h incubation in glucose-rich media were shifted to keratin or glucose (test and control, respectively) and forced to adapt to a stress-inducing environment.

After 96 h culture in keratin, *T. rubrum* had upregulated more cell wall-related genes than it had downregulated. The highest number of DEGs was observed at 96 h. Thus, *T. rubrum* endeavored to grow, develop, establish, and maintain cell wall integrity in the keratin-containing medium.

Among the genes repressed in keratin only two were also repressed in both the acriflavine and undecanoic acid treatments. They belonged to the stress-responsive GNAT acetyltransferase family. A gene encoding Wiskott-Aldrich syndrome protein family member 2 was also downregulated. It encodes a β-1,6-glucan putatively belonging to the KRE family. β-1,6-glucan integrates into the fungal cell wall, acts as the central core of the protein-carbohydrate network, interconnecting chitin and β-1,3-glucan, and associating mannoproteins there (Shahinian and Bussey, 2000). As this gene was repressed only after 96 h of fungal adaptation to keratin, the pathogen avoided excessive remodeling by restraining cell wall formation.

We observed the up-modulation of the hydrophobin gene, also modulated in response to acriflavine and undecanoic acid, attesting its role in the stress response of *T. rubrum*. Thus, the hydrophobin plays a role in the stress response of *T. rubrum*. The upregulation of hydrophobins in *T. rubrum* at all measurement time points of keratin incubation indicates that the pathogen tries to increase its cell wall hydrophobicity and, by extension, its virulence, during the early stages of host infection.

Genes encoding glycosyl hydrolase, glucanase, and chitinase were induced in response to keratin. These are the most commonly occurring hydrolases in the fungal cell wall. They are associated with cell wall polymer branching and cross-linking and the maintenance of cell wall plasticity during morphogenesis (Adams, 2004). They also influence fungal virulence as they participate in non-self cell wall degradation and enable the pathogen to penetrate and invade the host (Gruber and Seidl-Seiboth, 2012).

Keratin incubation also upregulated α-1,2-mannosyltransferase. In the human pathogenic mold *A. fumigatus*, deletion of an orthologous gene resulted in attenuated virulence. This gene is a potential target for novel antifungal therapies as it is unique in fungi (Wagener et al., 2008). The observed induction of α-1,2-mannosyltransferase in *T. rubrum* grown with keratin indicates that the fungus was trying to maintain its virulence and sustain its development under these conditions.

### Genes Involved in Hyphal Extension and Cell Growth Are Induced in *T. rubrum* Challenged With Cell Wall Stressors

Various stressors (CFW, CR, SDS, NaCl, KCl, and sorbitol) were added to the culture medium to determine their effects on radial colony growth (Figure 2). The fungal cell walls were sensitive to osmotic and cell wall stresses, and mycelial growth and development were inhibited. We then used qRT-PCR to evaluate the expression of genes involved in polarized growth, cell wall remodeling, and hyphal growth after exposure to the abovementioned cell wall stressors. The genes selected for evaluation are either regulated by the Ace2 transcription factor or belong to the RAM (regulation of Ace2 activity and cellular morphogenesis) signaling pathway. The RAM network is a protein kinase-signaling pathway associated to the maintenance of the cell wall integrity. RAM regulates the zinc finger transcriptional factor Ace2 which governs *fks*, *chs*, *cts*, and *scw11* genes, encoding 1,3-β-glucan synthase, chitin synthase, chitinase, and β-glucanase, respectively. These play vital roles in cell wall morphogenesis (Sbia et al., 2008; Saputo et al., 2012).

**FIGURE 4:**
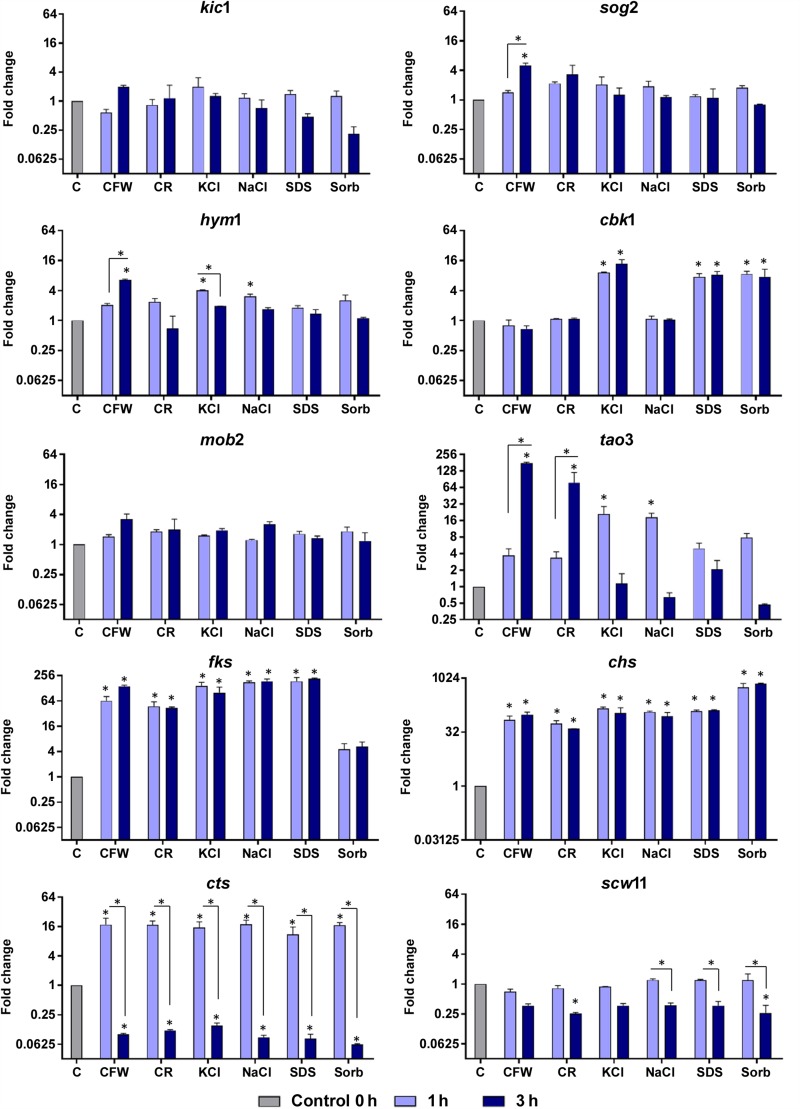
Relative expression levels of genes encoding components of the RAM network or putatively regulated by Ace2 after exposure of *T. rubrum* to Calcofluor White (CFW), Congo Red (CR), KCl, NaCl, SDS, and sorbitol (Sorb), for 1 or 3 h. Control (C) is the growth of *T. rubrum* for 96 h before addition of the stressors. Data are means and SD of three independent experiments. Asterisks indicate statistical significance determined by ANOVA followed by Tukey’s *ad hoc* test (*P* < 0.05).

Figure 4 shows that osmotic stressors did not affect the expression levels of the *mob2* and *kic1* genes. The leucine-rich repeat-containing *sog2* was induced only in response to CFW and *scw11* was repressed upon challenge with CR and sorbitol; both genes responded at the latest time of exposure. Saline stress induced an immediate response by the *hym1* gene and CFW exposure resulted in its induction after 3 h of. At both time points, *cbk1* responded to KCl, SDS, and sorbitol. The protein kinase *tao3* is strongly induced upon challenge with the cell wall stressors CFW and CR and is upmodulated in response to saline stress after 3h. The unique transcriptional pattern observed suggests that, although all these genes are directly related to cell wall integrity, as part of the RAM signaling network, their activation is time- and stress-responsive.

Fungal cell walls increase their chitin content in reaction to stress conditions (Walker et al., 2008; Fortwendel et al., 2009). This response is mediated by the upregulation of chitin synthases and/or the downregulation of chitinases (Heilmann et al., 2013). In the present study, we observed that *T. rubrum* induced *chs* and *fks1* and repressed *cts* following exposure to osmotic stressors (Figure 4). Cell wall restoration and selective modulatory activation of the RAM pathway genes are indicative of the efforts of *T. rubrum* to restore and maintain homeostasis in response to external stressors.

### Cell Wall, Virulence, and Antifungal Drug Development

Increasing global transcriptome data favors more robust studies on fungal gene modulation that may elucidate fungal metabolism, pathogenesis, and drug resistance. The results obtained herein may also afford strategies for the identification of novel drug development targets.

Here, five genes were modulated in *T. rubrum* in response to the various stressors. Three belonged to the GNAT family, one was a class V chitinase, and the fifth was a hydrophobin. The observed stress-mediated response of the hydrophobin gene suggests its possible use as a therapeutic antifungal drug target. Antifungal targets must be essential proteins, have a high degree of sequential similarity across pathogenic fungal species, and be absent in the human genome (Liu et al., 2006). Hydrophobins present with low overall sequence conservation. Nevertheless, they have a broad range of functions including the regulation of cell wall integrity. They can also change the hydrophilic/hydrophobic properties of cell surfaces and are unique to filamentous fungi (Wosten, 2001; Linder et al., 2005; Bayry et al., 2012).

The GNAT superfamily is ubiquitous across many taxa, including humans and fungi. However, a few of its members are potential antifungal drug targets. Identification of the structural differences between human and fungal GNAT members may facilitate selective antifungal drug design (Masubuchi et al., 2003; Hurtado-Guerrero et al., 2008).

A class V chitinase was repressed in response to acriflavine and undecanoic acid exposure but induced in the presence of keratin. Thus, it plays a vital role in substrate adaptation. It was reported that in *T. rubrum* this gene participates in keratin degradation (Lopes et al., 2019). Fungal cell walls contain high levels of chitin and they are cleaved by chitinases during cell wall remodeling. Thus, disruption of this process is expected to affect fungal virulence and survival and points to chitinases as antifungal drug targets (Rush et al., 2010).

## Conclusion

Environmental changes and external stresses induce continuous remodeling of fungal cell walls, and the transcriptional responses of *T. rubrum* to those observed in this study are essential for its resistance and pathogenicity. We have generated a robust dataset, which underscores the relevance of the cell wall in pathogen-host interactions, advancing our knowledge on the cell wall regulatory mechanisms of dermatophyte fungi. Our results indicate that stress conditions forced *T. rubrum* to restructure its cell wall by modifying the cell wall composition; thus, affecting fungal virulence. The modulation of genes controlling the fungal cell-wall structure has great potential in the identification of putative targets for the development of novel antifungal drug therapies.

## Data Availability Statement

The RNA-seq data is available at the GEO database under the accession number GSE134406.

## Author Contributions

MM and LGS drafted the manuscript. MM, LGS, and LDS performed the experiments. PS performed the computational analyses. AR and NM-R supervised the study and prepared the manuscript. NM-R designed the project. All authors participated in the data analysis and critical revision of the manuscript, and approved the final version.

## Conflict of Interest

The authors declare that the research was conducted in the absence of any commercial or financial relationships that could be construed as a potential conflict of interest.

## Footnotes

1 ftp://ftp.broadinstitute.org/pub/annotation/fungi
