# Supplementary material for: Global Analysis of Cell Wall Genes Revealed Putative Virulence Factors in the Dermatophyte Trichophyton rubrum
Source: Front Microbiol. 2019 Sep 19;10:2168. doi: 10.3389/fmicb.2019.02168 (PMC6761320; doi:10.3389/fmicb.2019.02168)
Supplement: Supplementary file 1 [file Table_1.pdf]

Supplementary Table S1. Cell wall-related genes of *T. rubrum* modulated at various time points in response to exposures to acriflavine (ACF), undecanoic acid (UDA), or keratin.

ID	ACF vs. control			UDA vs. control		Keratin vs. control			Gene Product Name
	3 h	12 h	24 h	3 h	12 h	24 h	48 h	96 h	
**Genes modulated in response to exposure of ACF, UDA and keratin**									
TERG_02517	-3.70	-5.45	-12.32	-	-2.41	-	1.72	-	N-acetyltransferase, GNAT family, putative (*A. benhamiae*)
TERG_04234	-	1.79	2.24	-5.14	-1.93	4.10	3.49	3.30	hydrophobin, putative (*T. verrucosum*)
TERG_05545	-2.86	-4.39	-10.50	-2.82	-1.75	-	-	-1.74	GNAT family acetyltransferase, putative (*A. benhamiae*)
TERG_05625	-2.09	-1.97	-2.15	-2.35	-2.57	3.95	-	2.44	class V chitinase, putative (*T. verrucosum*)
TERG_07408	-2.51	-3.14	-3.38	-1.98	-1.63	-2.62	-2.32	-2.98	acetyltransferase, GNAT family (*A. benhamiae*)
**Genes modulated only in response to exposure of ACF and UDA**									
TERG_01252	1.70	2.45	2.80	-	2.37				catalase A
TERG_03896	-	1.78	-	-	1.68				mannose-6-phosphate isomerase
TERG_07456	1.68	-	-	-2.90	-				cell wall protein PhiA (*T. equinum*)
**Genes modulated only in response to exposure of ACF and keratin**									
TERG_01507	2.37	2.29	-			-	-	2.19	extracellular proline-glycine rich protein (*T. verrucosum*)
TERG_08286	1.90	1.96	2.60			-	-	2.38	GPI anchored CFEM domain protein (*T. verrucosum*)
**Genes modulated only in response to exposure of UDA and keratin**									
TERG_00060				-	1.72	-	-	3.52	GPI anchored cell wall protein, putative (*A. benhamiae*)
TERG_00216				2.88	-	1.90	-	-	endochitinase (*T. equinum*)
TERG_03624				-2.88	-	2.05	1.90	1.99	SUN domain protein (Uth1), putative (*T. verrucosum*)
TERG_03963				-	-1.73	1.53	2.14	2.24	mannosyl phosphorylinositol ceramide synthase SUR1 (*T. equinum*)
TERG_05576				-1.75	-	-	1.76	2.60	cell wall glucanase (Scw11), putative (*A. benhamiae*)
TERG_05626				-1.96	-	2.25	-	3.17	chitinase (*T. equinum*)
TERG_08225				-2.01	-	-	-	1.50	hypothetical protein
TERG_08969				-	2.46	2.03	1.82	1.66	cytosolic Cu/Zn superoxide dismutase, putative (*A. benhamiae*)
**Genes modulated only in response to exposure of ACF**									
TERG_00250	1.62	1.78	-						*N*-acetylglucosamine-phosphate mutase (*T. equinum*)
TERG_00625	-	-	1.59						glycolipid anchored surface protein (GAS1) (*T. equinum*)
TERG_01127	-	-	1.54						1,3-beta-glucan synthase component FKS1
TERG_03618	1.61	-	-						class V chitinase, putative (*T. verrucosum*)
TERG_06053	-	8.37	-						catalase (*T. tonsurans*)
TERG_07734	-2.47	-4.04	-3.93						O-methyltransferase, putative (*T. verrucosum*)

**Genes modulated only in response to exposure of UDA**						
TERG_00481	-2.56	-				beta-glucosidase, putative (*T. verrucosum*)
TERG_00707	-1.82	-				GPI anchored serine-threonine rich protein (*T. verrucosum*)
TERG_01869	-3.06	-				TOS1 (*T. equinum*)
TERG_02350	-	-1.99				endochitinase (*T. equinum*)
TERG_06016	1.81	-				glycosyl hydrolase (*T. tonsurans*)
TERG_06144	-3.68	-				cell wall serine-threonine-rich galactomannoprotein Mp1 (*A. benhamiae*)
TERG_07269	-1.75	-				mannose-1-phosphate guanyltransferase
TERG_07641	-1.60	-				GPI-anchored cell wall protein Pst1, putative (*A. benhamiae*)
TERG_11951	-2.67	-				prp 6 CRoW domain-containing protein (*M. canis*)
TERG_12281	1.95	-				glycosyl hydrolase (*T. tonsurans*)
TERG_12282	2.16	-				glycosyl hydrolase (*T. tonsurans*)
**Genes modulated only in response to exposure of keratin**						
TERG_00218			-	-	2.59	C2H2 transcription factor (Swi5), putative (*A. benhamiae*)
TERG_00342			-1.98	-1.56	-	phosphoglucomutase
TERG_00638			2.17	1.98	1.96	cellobiose dehydrogenase, putative (*A. benhamiae*)
TERG_00693			-	-	-1.70	wiskott-Aldrich syndrome protein family member 2 (*M. canis*)
TERG_01464			1.71	-	-	bifunctional catalase-peroxidase Cat2 (*T. verrucosum*)
TERG_01837			2.17	3.48	2.39	glycosyl hydrolase (*T. equinum*)
TERG_01917			-	-	3.30	O-methyltransferase, putative (*A. benhamiae*)
TERG_01967			-	-	2.87	GPI anchored protein, putative (*T. verrucosum*)
TERG_02005			-	-	1.84	catalase (*T. tonsurans*)
TERG_02705			2.77	3.69	2.74	class III chitinase (*T. tonsurans*)
TERG_02719			3.41	3.27	4.52	glycosyl hydrolase (*T. equinum*)
TERG_02742			2.28	2.41	1.89	glycosyl hydrolase (*T. tonsurans*)
TERG_02791			-	-3.01	-3.35	cell wall integrity signaling protein Lsp1/Pil1 (*T. equinum*)
TERG_02973			-	-	1.84	morphogenesis protein (Msb1), putative (*T. verrucosum*)
TERG_03144			-1.65	-1.59	-	glucose-6-phosphate isomerase
TERG_03223			6.07	6.58	5.95	N-acetylglucosamine-6-phosphate deacetylase
TERG_03226			4.92	5.62	5.67	glucosamine-6-phosphate deaminase
TERG_03353			-	-	-1.77	endoglucanase (*T. equinum*)
TERG_03379			-	1.53	-	AGC/NDR/NDR protein kinase
TERG_03398			-	-	-2.20	chitinase (*M. canis*)
TERG_03489			-	-	3.05	hypothetical protein
TERG_03620			2.91	2.40	2.08	glycosyl transferase (*T. tonsurans*)
TERG_03729			2.89	3.14	3.25	6-phosphogluconate dehydrogenase (decarboxylating)
TERG_03854			-	-	-1.61	laccase (*T. tonsurans*)

TERG_04564	1.91	1.78	-	mixed-linked glucanase (*T. equinum*)
TERG_04887	1.79	2.09	1.68	endo-1,3-beta-glucanase (*T. equinum*)
TERG_05002	-1.85	-1.57	-	UDP-glucose 4-epimerase GalE
TERG_05530	-	-	2.14	glycosyl hydrolase, putative (*A. benhamiae*)
TERG_05618	-1.83	-	-	Lcc2 (*T. equinum*)
TERG_05879	-	-1.54	-1.60	GPI mannosyltransferase (*T. tonsurans*)
TERG_06242	5.67	5.84	6.73	glucanase, putative (*T. verrucosum*)
TERG_06397	2.07	-	1.65	alpha-1,2-mannosyltransferase (*T. equinum*)
TERG_06638	-	2.89	6.00	endochitinase (*T. equinum*)
TERG_06749	-1.62	-	-	cell wall protein, putative (*A. benhamiae*)
TERG_06925	-	-	1.59	class V chitinase (*T. tonsurans*)
TERG_06929	2.76	4.05	2.27	chitinase (*T. equinum*)
TERG_06986	4.05	4.56	3.77	exo-beta-1,3-glucanase, putative (*A. benhamiae*)
TERG_07406	3.89	4.12	3.74	alpha-mannosidase (*T. tonsurans*)
TERG_07597	-	2.05	1.82	SOK1 (*T. equinum*)
TERG_07657	-	-	1.91	chitin synthase class VI (*T. equinum*)
TERG_07662	-	-	-2.08	mannosylphosphate transferase (*T. tonsurans*)
TERG_07817	-	2.33	-	endoglucanase (*T. equinum*)
TERG_07987	-	-	-3.45	GNAT family acetyltransferase (*T. equinum*)
TERG_08058	-	2.44	3.41	alpha-1,2-mannosidase family protein (*T. verrucosum*)
TERG_08191	-	-1.58	-1.67	glucooligosaccharide oxidase (*T. equinum*)
TERG_08211	-2.62	-	-2.41	acetyltransferase, GNAT family, putative (*A. benhamiae*)
TERG_08498	-2.65	-2.57	-1.94	triosephosphate isomerase
TERG_08882	-	-	2.54	C2H2 transcription factor (Crea), putative (*T. verrucosum*)
TERG_12107	-2.45	-1.99	-1.61	1,4-alpha-glucan-branching enzyme
TERG_12108	-2.40	-1.88	-	1,4-alpha-glucan-branching enzyme
TERG_12318	-	-	1.67	chitin synthase (*T. tonsurans*)
TERG_12319	-	-	1.68	chitin synthase 2

(-) not modulated at the time point
