# Supplementary material for: Global Analysis of Cell Wall Genes Revealed Putative Virulence Factors in the Dermatophyte Trichophyton rubrum
Source: Front Microbiol. 2019 Sep 19;10:2168. doi: 10.3389/fmicb.2019.02168 (PMC6761320; doi:10.3389/fmicb.2019.02168)
Supplement: Supplementary file 2 [file Table_2.pdf]

Supplementary Table S2. List of primers used in qRT-PCR analysis.

ID	Gene Product Name	Primers (5' - 3')	Efficiency (%)	Concentration (nM)
TERG_00216	endochitinase *(T. equinum)*	FW: TCGACAACGGTCTAGGGAGGT<br>REV: CCTCGACGAAGGTAGCAATC	102.30	700
TERG_00759	conidiophore development protein *hym1* (*T. violaceum*)	FW: GCGTACGTGGACAGTGGTGAT<br>REV: GCGGTCGTCTCGAAGCA	105.94	400
TERG_01127	1,3-beta-glucan synthase component FKS1	FW: TTGACTGAGCGTGGTTTCTG<br>REV: GGCGTAGATCTGACACACGA	98.35	400
TERG_01721	STE/STE20/YSK protein kinase *kic1*	FW: AGCGGGCAGGTCAAGCT<br>REV: TGGTCATTGTAGCGGAAAGTTG	102.20	400
TERG_01788	cell morphogenesis protein *tao3* (*T. equinum*)	FW: CCGGGTGGTAGACGAACCT<br>REV: CGTACTAACTGGGTGATTGGTTCTG	96.35	500
TERG_02705	class III chitinase (*T. tonsurans*)	FW: CAAGGAGATGCTGGAAGGAG<br>REV: AGCCGAGGACGAAGTTGTTA	101.48	400
TERG_02719	glycosyl hydrolase (*T. equinum*)	FW: AGCCCTTCTTCCTCATCCTC<br>REV: GGTCGGGTAGAACTTGTCCA	103.95	300
TERG_02863	protein kinase activator *mob2* (*T. equinum*)	FW: CAGCCCCTCATCGATATCTACAC<br>REV: ACCGGCAAGGGCTTCAG	102.88	400
TERG_03379	AGC/NDR/NDR protein kinase *cbk1*	FW: AAATTGTCAAGAAGGCCACCTGCG<br>REV: TGGAAGCTCGAAATGAGCGGTACA	95.93	200
TERG_03963	mannosyl phosphorylinositol ceramide synthase SUR1 (*T. equinum*)	FW: AGCTGTACGACACAACGGAAT<br>REV: ATACAGCCATGAAAGGACTGC	105.40	200
TERG_04234	hydrophobin, putative (*T. verrucosum*)	FW: GGCATACATCTTGGTGGTTTC<br>REV: CAGACAGTGGAGGTGGATGTT	95.09	400
TERG_04564	mixed-linked glucanase (*T. equinum*)	FW: TCACTACGGAGTGCAAGACG<br>REV: TTGGCTCCTGGTAGACGTTC	96.76	300
TERG_05576	cell wall glucanase (Scw11), putative (*A. benhamiae*)	FW: CCCGACTCCAGGAACTTACA<br>REV: GAAGGACAGACGGTTGCACT	98.00	500
TERG_07599	cell morphogenesis protein Sog2 (*T. tonsurans*)	FW: TCCTGGAAATCCTCGACCTAAG<br>REV: GCGGACTTCCTCAGGCAAT	101.34	400
TERG_08191	glucooligosaccharide oxidase (*T. equinum*)	FW: CAGCTCAATGCTTTTGGAGAG<br>REV: TCTCCGTCTACCATGGACTGT	91.90	300
TERG_12319	chitin synthase 2 *chs*	F: AGCCAACTGCCTTGTACCAT<br>R: GTAATCCGACCCATCCCTTT	90.29	600
TERG_04402	glyceraldehyde 3-phosphate dehydrogenase	FW: GCGTGACCCAGCCAACA<br>REV: CGGTGGACTCGACGATGTAGT	99.90	200
TERG_05742	DNA-dependent RNA polymerase II	FW: TGCAGGAGCTGGTGGAAGA<br>REV: GCTGGGAGGTACTGTTTGATCAA	94.99	300
