# Supplementary material for: Global Analysis of Cell Wall Genes Revealed Putative Virulence Factors in the Dermatophyte Trichophyton rubrum
Source: Front Microbiol. 2019 Sep 19;10:2168. doi: 10.3389/fmicb.2019.02168 (PMC6761320; doi:10.3389/fmicb.2019.02168)
Supplement: Supplementary file 3 [file Table_3.pdf]

Supplementary Table S3. RNA-seq reads mapped to the *T. rubrum* reference genome.

Sample	Raw reads	High-quality reads	Mapped reads STAR	Total mapped reads (%)
Control 24 hours I	10,298,058	10,079,869	9,513,704	94.38
Control 24 hours II	11,730,284	11,470,387	9,274,834	80.86
Control 24 hours III	13,134,053	12,870,769	12,130,966	94.25
Control 48 hours I	12,139,571	11,873,826	11,045,360	93.02
Control 48 hours II	8,484,471	8,220,328	7,582,730	92.24
Control 48 hours III	13,900,719	13,563,244	12,759,015	94.07
Control 96 hours I	10,846,570	10,620,463	9,913,587	93.34
Control 96 hours II	9,950,533	9,707,227	9,214,841	94.93
Control 96 hours III	12,788,333	12,287,043	11,533,469	93.87
Keratin 24 hours I	9,586,259	9,339,491	7,040,104	75.38
Keratin 24 hours II	10,093,406	9,815,286	8,266,023	84.22
Keratin 24 hours III	13,910,853	13,576,336	11,848,476	87.27
Keratin 48 hours I	11,636,035	11,292,471	9,374,940	83.02
Keratin 48 hours II	9,081,225	8,769,772	6,548,726	74.67
Keratin 48 hours III	10,036,969	9,786,133	8,751,212	89.42
Keratin 96 hours I	8,644,359	8,369,738	6,533,731	78.06
Keratin 96 hours II	8,440,082	7,993,994	6,216,676	77.77
Keratin 96 hours III	12,753,703	12,436,022	9,652,577	77.62
